# Gain Curves, Reproductive Efficiency, and Sex Allocation

**DOI:** 10.1002/ece3.73539

**Published:** 2026-05-17

**Authors:** Martin Burd

**Affiliations:** ^1^ Royal Botanic Gardens Victoria Melbourne Victoria Australia

**Keywords:** evolutionarily stable strategy, frequency‐dependent selection, gain curve, local mating competition, pollen‐ovule ratio, pollination efficiency, Shaw‐Mohler equation

## Abstract

Reproductive efficiency reflects the gap between an organism's potential success and its actual achievement. Exploring how theoretical sex allocation models deal with reproductive efficiency reveals shortcomings in two fundamental elements of the theory, fitness gain curves and the Shaw‐Mohler equation. Gain curves depict success but make no reference to the production of the entities that accrue fitness, so they imply nothing about efficiency in its common sense, the fraction of successful reproductive entities in relation to the number produced. Because the number of successful entities cannot exceed the number produced, a gain curve implies a minimum allowable production, but the implication itself exposes new problems. Gain curves are used to replace terms in the Shaw‐Mohler equation that were meant to represent an individual's fair competitive share of total population fitness. If gain curves themselves represent individual fitness outcomes, the theoretical solutions can imply unequal aggregate success of male and female mating agents, a biological impossibility in a sexually reproducing population. If gain curves represent inputs to an arena of mating interactions, the theoretical solutions can imply misleading or biologically impossible patterns of reproductive efficiency. The relationship between pollination efficiency and pollen‐ovule ratios in flowering plants is used as a window into the shortcomings of gain curves and the Shaw‐Mohler equation. There are new modeling approaches to sex allocation that are more explicit about the processes that lead from production of male and female reproductive entities to their eventual fitness outcomes.

## Graphical abstract

Exploring the concept of reproductive efficiency reveals several shortcomings in fitness gain curves and the Shaw‐Mohler equation for sex ratio evolution. Gain curves that refer to the gamete inputs to mating interactions make no statement about the production of gametes. Gain curves that refer to the fitness outcomes of mating often violate the required equality of total male and female fitness in a population. The example of pollination efficiency in flowering plants is used as a window onto these and other conceptual difficulties of sex allocation models based on the Shaw‐Mohler equation.

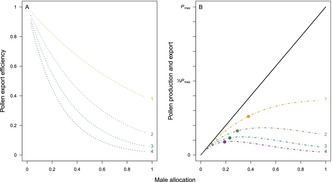

## 1 Introduction

A notable feature of sexual reproduction is the abundance of gametes (or other male and female entities) that take part and the seeming inefficiency of their interaction. This article examines reproductive efficiency, particularly pollination efficiency in angiosperms, as a window into the nature of two mathematical tools that have become foundations of sex allocation theory, the Shaw‐Mohler equation and gain curves (Shaw and Mohler 1953; Charnov 1979, 1982).

Gain curves are functions describing the effect of reproductive investment on male or female success (Charnov 1979, 1982). Although they have been used in theoretical models for decades, gain curves easily violate the so‐called Fisher condition (Burd 2026), the requirement that aggregate male fitness in a sexually reproducing population equal the aggregate female fitness (Houston and McNamara 2005). The Fisher condition follows directly from the biology of syngamy. Failure to comply with the Fisher condition is a serious failure in any model involving sexual reproduction.

The Shaw‐Mohler equation, as well, has limitations that have largely gone unrecognized (Burd 2024). Most importantly, the locus of fitness in this equation was unstated and implicit in the original formulation. This absence hides the lack of representation within the Shaw‐Mohler equation of processes that transform inputs to a mating arena into fitness outcomes, the core of reproductive efficiency.

## 2 The Shaw‐Mohler Equation

Shaw and Mohler (1953) presented the fitness of a focal parent *i* as a function of the number of sons, *m*_*i*_, and daughters, *f*_*i*_, it produced, relative to the total number of breeding males, *M*, and females, *F*, in the population:

(1) $$w_{i}=\frac{m_{i}}{M}+\frac{f_{i}}{F}.$$

The ratios in Equation (1) represent *i*'s fair competitive share of whatever total population fitness the breeding individuals attain, on the assumptions of panmixia and equal chance of success for all males and females in the population. Shaw and Mohler (1953) did not specify population fitness in their equation, thus implicitly setting its magnitude to 1, which is acceptable in that fitness can be measured in arbitrary units. However, it had the effect of obscuring where fitness lay in the equation. Population fitness, *W*, can be made explicit:

(2) $$w_{i}=\left(\frac{m_{i}}{M}+\frac{f_{i}}{F}\right)W.$$

It is now clear that *i*'s male fitness is (*m*_*i*_/*M*)*W* and its female fitness is (*f*_*i*_/*F*)*W*. The terms *m*_*i*_, *M*, *f*_*i*_, and *F* do not themselves represent fitness.

Even with an explicit term for total fitness, the Shaw‐Mohler equation lacks any explanation of how the interactions among *M* and *F* mating agents lead to *W*. Thus, the competitive share ratios in the Shaw‐Mohler equation capture the effect of frequency‐dependent selection, but not the source of total fitness (Burd 2024). Inasmuch as reproductive efficiency lies in the translation of the numbers of mating agents into the numbers that are successful (their fitness attainment), this structural limitation of the Shaw‐Mohler equation will have consequences seen below.

## 3 What Do Gain Curves Gain?

At issue is whether a gain curve represents the inputs to a reproductive process like mating, or the outcomes of that process. At their introduction, gain curves seem to have been intended to represent fitness outcomes: ‘the hermaphrodite has a proportion *f* of a female's fitness (through seeds) and a proportion *m* of a male's fitness through pollen. Factors, such as the ability to attract vectors to carry pollen or seeds away (in addition to the actual number of gametes), affect the chances of an individual to gain reproductive success via sperm or eggs’ (Charnov 1982, 220). Charnov referred to ‘fitness,’ and the clarification ‘in addition to the actual number of gametes’ puts the idea in the realm of fitness outcomes rather than solely gamete inputs. Thereafter, most theoretical models and interpretations of empirical patterns of sex allocation referred unambiguously to gain curves in terms of fitness gains (e.g., Lloyd 1984; Brunet 1992; Campbell 2000; Klinkhamer and de Jong 2002; Baeza 2007; Wiernasz and Cole 2009). Empirical estimations of gain curves have also usually involved measurement of fitness outcomes (Emms 1996; Emms et al. 1997; McCartney 1997; Campbell 1998; Yund 1998; Johnson and Yund 2009; Aljiboury and Friedman 2022; Chen and Pannell 2025). This appears to be the common interpretation of the gain in ‘gain curve.’

Pen and Weissing (2002, 33), however, noted some flexibility in meaning: ‘The term *m*(*x*) represents the returns on investment in sons, as determined by the mutant's behaviour *x*, and *f*(*x*) the returns on investment in daughters. “Returns on investment” seems rather vague, but this is because its interpretation may vary from one model to another. Often it simply means “number of individuals that survive until adulthood”.’ The continuum from production of mating agents through their entry to a mating arena to the ultimate fitness they attain provides leeway to vary the point at which a ‘gain’ is declared. For example, Rademaker and de Jong (1998) interpreted gain in terms of fitness outcomes, but measured the transfer of fluorescent dyes from anthers to the stigmas of recipient flowers as a proxy for pollen transfer. Stigmatic pollen deposition lies somewhere between entry to the pollination environment and final male fitness. The model of Harder and Johnson (2025) represented ‘the proportion of pollen exported to other individuals’, wording that would also seem to lie short of final fitness accrual.

Whatever meaning is intended, it should be transparent. And yet ambiguity was present from the earliest uses of gain curves. Charlesworth and Charlesworth (1981) defined functions for male and female ‘fertility’ with the specification that progeny of the female are counted ‘only if they survive to maturity.’ Here, ‘fertility’ seems clearly to mean ultimate fitness, at least for female function. But in their fitness equation, male fitness through outcrossing for individual *i* is specified by the term (*b*_*i*_/*b*)*U*, in which *U* is the mean female ‘fertility’ of individual plants in the population. The ratio *b*_*i*_/*b* defines *i*'s fair competitive share of *U* relative to the mean male ‘fertility’ *b*. This ratio makes sense only if *b*_*i*_ and *b* refer to pollen *inputs* to competition and mating. Thus, Charlesworth and Charlesworth (1981) defined female fertility in a way that implies fitness outcome, but operationally defined male fertility in a way that should mean pollen input.

Morgan (1992, 1201) also seems to have combined an outcome definition for female gains with an input definition for male gains. The female gain function described ‘the relationship between allocation and the number of ovules fertilized’ while the relationship for male gains was ‘between allocation and number of pollen grains successfully disseminated’. Morgan's (1992) further descriptions of the exponents in the male gain function refer only to ‘pollen production’ and pollen ‘successfully deposited on the pollinator's body.’ Friedman and Barrett (2009) also described male and female gains at different points along the production‐to‐fitness continuum. They outlined a model structure in which female fitness ‘is proportional to resources invested in ovules (*F*) multiplied by the fraction of ovules that are fertilized’, while male fitness ‘is proportional to the resources invested in pollen (*M*), the pollen removed by insects (*p*)…and the pollen removed by wind…’ (Friedman and Barrett 2009, 1516–1517). Male gains seem to stop short of fertilization success in these uses of male gain curves.

Are the differences in wording surrounding gain curves merely linguistic adornment? The mathematical operations may be unaffected, but the biological insight we wish to extract from gain curves depends entirely on what they mean. If gain curves represent fitness, they face a compositional problem. An individual's pattern of fitness gain in response to its own sex allocation, considered in isolation, will not be the pattern all individuals experience when all individuals are subject to the same change in sex allocation under the force of selection (Burd 2026). In particular, monotonic gain curves can exist only for individuals in a non‐evolving background of abundant mating opportunities. Moreover, fitness gain curves must satisfy the equality of total male and female fitness in a population, the Fisher condition. This requirement poses a serious challenge (Burd 2024). On the other hand, if gain curves represent inputs, they fit more easily into the structure of the Shaw‐Mohler equation, but we must accept the limitations of that equation as a modeling tool.

## 4 What Does Efficiency Mean?

Like ‘gain curve,’ this word can also have multiple meanings, an imprecision of terminology faced by economists, as well (Sickles and Zelenyuk 2019). A simple operational definition arises from the way reproductive efficiency is measured. Efficiency is simply the fraction of sperm or eggs (or pollen or ovules, etc.) produced by an organism that is ultimately successful at some later stage in the process of reproduction. Measurement of this fraction is often as elementary as counting the number of eggs and the number of embryos or larvae that later develop from them (Brazeau and Lasker 1992; Manríquez and Castilla 2010; Fan et al. 2013). In flowering plants, the fertilization efficiency of ovules can be detected by the presence of a pollen tube (Snider et al. 2009) or the formation of seeds (Burd 1994a). Efficiency of male function can be measured even more simply. The mean number of pollen grains counted on the stigmas of flowers relative to the mean number of grains produced per flower provides a population‐wide estimate of pollination efficiency (Gong and Huang 2014). Even when the word ‘efficiency’ is not used, terms like ‘fertilization success rate’ mean the same thing (Polak and Simmons 2009). Efficiency in this usage is a dimensionless measure, a fraction of success out of a starting number or amount. The term *fractional efficiency* might be used to specify this meaning.

Attention to definition is important because ‘efficiency’ can be used to describe other rates with particular dimensions. Damselflies gather at ponds and lake margins where mating and oviposition occur, and Fincke (1986, 794) observed these behaviors and defined efficiency as follows: ‘Mating efficiency was measured directly as the number of matings leading to ovipositions per visit to the pond. This measure was compared to mating efficiency calculated by dividing total mates by days alive (mates/day).’ The latter metric carries units and so is clearly different from fractional efficiency. Yet another meaning of efficiency relevant to gain curves is *marginal efficiency*, the rate of incremental fitness gain from incremental resource investment at any particular point along the curve. This meaning of efficiency was employed by Lloyd (1983, 1984), and by Harder and Johnson (2025). These uses of marginal fitness are examined below.

**FIGURE 1:**
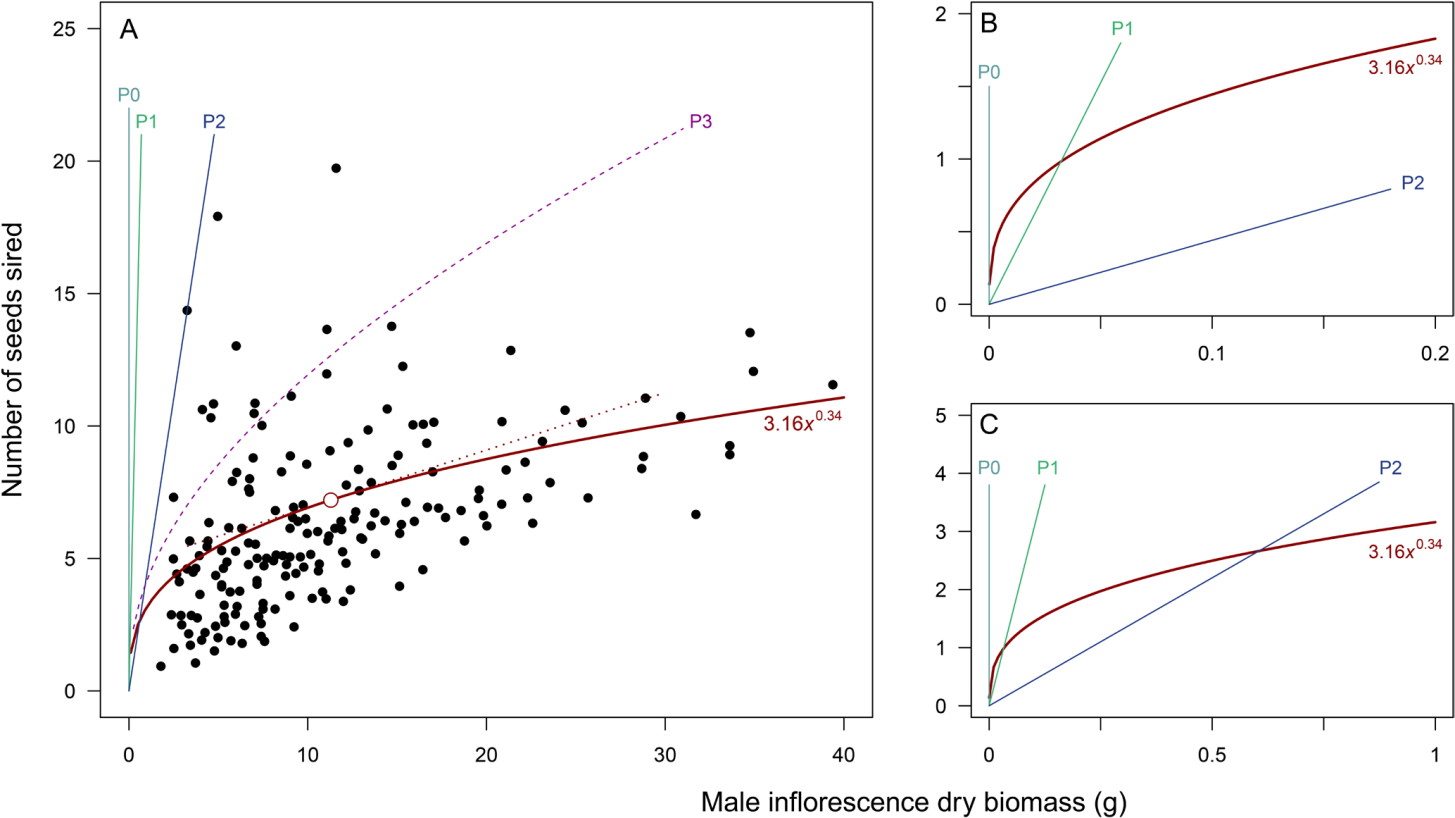
Example of empirical determination of marginal efficiency and implied pollen production of a male gain curve. (A) Filled circles: Paternity estimates for *Ambrosia artemisiifolia* plants, from figure 2b of Aljiboury and Friedman (2022). Continuous, heavy red curve: Gain curve given by (number of seeds sired) = 3.16·(male inflorescence mass in g)^0.34^. Open circle on gain curve: Value at the mean inflorescence mass in the sample, 11.3 g. Dotted red line: Tangent to the gain curve at the mean male inflorescence mass. Line P0: Pollen production sufficient to allow the entire positive extent of the gain curve. Line P1: Allowable pollen production for any part of the gain curve equal to or exceeding 1 seed sired. Line P2: Allowable pollen production for all the empirical data in the sample. (Allowable production lines ensure that the number of pollen grains equals or exceeds the number of seeds sired. See text for further explanation.) Dashed curve P3: A nonlinear pollen production pattern that implies diseconomies of scale. (B, C) Details of panel A showing portions of the gain curve exceeding P1 or P2. Note the different scaling of axes.

I know of no empirical estimation of marginal efficiency of a gain curve for any species, but it will be helpful to consider how it would be done. A gain curve must first be estimated. For example, Aljiboury and Friedman (2022) used paternity analysis to determine the number of seeds sired by individual *Ambrosia artemisiifolia* plants whose male investment had been manipulated by removal of inflorescence branches. Their results are shown in Figure 1A with a gain curve given by (number of seeds sired) = 3.16·(dry biomass of male inflorescences in g)^0.34^. (This function corresponds to the curve illustrated in figure 2b of Aljiboury and Friedman (2022), although they report an exponent of 0.85 for the gain curve they calculated from these data.) Marginal efficiency is indicated by the slope of a line tangent to the gain curve at any given point, that is, by the first derivative of the gain curve function. A convenient point for illustration is the mean male inflorescence mass in the sample, 11.3 g, shown as an open symbol on the gain curve in Figure 1. The tangent at this point has a slope (marginal efficiency) of approximately 0.22 seeds sired per gram of male inflorescence.

**FIGURE 2:**
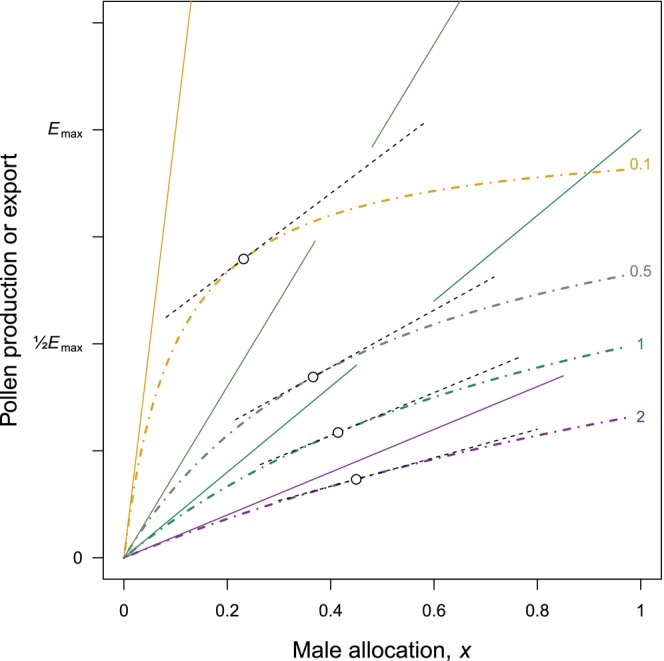
Pollen export *E*, marginal efficiency, and implied pollen production in the model of Harder and Johnson (2025). Interrupted curves: Pollen export function *E* = *E*_max_ *x*/(*x* + *d*) for values of parameter *d* indicated to the right of each curve. Open circles: Pollen export at the ESS allocation for each curve. Dashed lines tangential to the export curves: Visualization of marginal efficiency of each export curve at its ESS. Continuous lines: Implied pollen production with slope *E*_max_/*d* for each export curve (lines interrupted for clarity to show ESS and tangents for other curves). See text for interpretation.

How does marginal efficiency of a gain curve relate to fractional efficiency? As the derivative of a gain curve function, marginal efficiency depends solely on the gain curve, whether it is estimated empirically or asserted theoretically. Fractional efficiency, in contrast, depends on the difference between the successes represented by a gain curve and the number of initial attempts. In Figure 1A, for example, fractional efficiency would depend on the number of pollen grains produced at any given male biomass and the average number of sirings represented on the gain curve. Pollen counts as a function of male biomass could, of course, be measured directly for *A. artemisiifolia* . Often, however, we are dealing with theoretically postulated gain curves, so it will be helpful to consider what a gain curve per se implies about pollen production.

## 5 Gain Curves and Production Functions

Fitness in a sexual life cycle rests ultimately on successful formation of new zygotes. A gain curve that represents fitness, even if measured on an arbitrary scale, such as a minimum of 0 and a maximum of 1, can therefore be translated to a number of zygotes (or offspring at a later stage of development). A simple biological truth is that a gain curve cannot attribute parentage of more zygotes than the original number of sperm, eggs, pollen, ovules, or other relevant entity that an individual produced.

The male gain curve in Figure 1A represents the number of seeds sired. A useful reference point occurs where the gain curve specifies fertilization of 1 seed, which occurs at a dry biomass of 0.033 g. A plant must—at an absolute minimum—have produced at least 1 pollen grain at this point, implying a minimum production rate of 1/0.033 pollen grains per gram dry mass of male inflorescences. If the cost of pollen (and the floral structures needed for its production and dispersal) is constant, the number of pollen grains produced will be a linear function of resource investment. The minimum linear function that accounts for the siring of 1 seed is shown as P1 in Figure 1A. Extending P1 to 40 g of male dry biomass would imply production of about 1200 pollen grains, while a single anther of *A. artemisiifolia* actually produces a mean of more than 3000 (Šaulienė et al. 2012). P1 is not, therefore, realistic, but it is a conceptual minimum.

An alternative function, shown as line P2 in Figure 1A is the minimum production that accounts for all the empirical data from which the gain curve is estimated.

A conceptual shortcoming of P1 and P2 is that they leave a small part of the gain curve lying above the pollen production line, implying that a plant with this small male investment would sire more seeds than the number of pollen grains it produced (Figure 1B,C). The only line that lies everywhere above the gain curve in Figure 1A is P0, which implies an infinite rate of conversion of resource investment into pollen grains. A similar implication arises for any power‐function gain curve with an exponent less than 1.

Economies or diseconomies of scale, in which the unit cost of a pollen grain varies with the amount of pollen produced, would yield nonlinear production functions. Nonlinear functions, such as P3 in Figure 1A, could lie everywhere above a power‐function gain curve without implying an infinite rate of production. P3 represents a diseconomy of scale: the rate of production declines with higher and higher levels of production. Diseconomies of scale for production are difficult to understand in biological terms. In economics, diseconomies of scale typically arise in very large enterprises that have exhausted earlier economies of scale in their output and now face supply chain or managerial constraints (Canbäck et al. 2006). It is not obvious what the biological equivalent would be. Economies of scale are more biologically plausible, as a consequence of fixed costs of reproduction. If, for example, pollen dispersal depends on a tall scape to display an inflorescence, the cost of the scape will yield a very high cost per pollen grain if only a few grains are produced, but a lower cost per pollen grain if many are produced.

The anomalies presented by P0, P1, P2, and P3 might be dismissed as irrelevant. They arise principally for small numbers of seeds and pollen grains, possibly fewer than 1. The point is not that we must seriously consider any of these production functions, but to show that a gain curve implies *some* level of production. Yet a gain curve on its own, whether postulated theoretically or measured empirically, provides rather limited information. Inferring production from a gain curve is fraught with conceptual pitfalls. A simpler approach, used below in section 9, is to establish a production function and an efficiency function a priori, and obtain a gain curve from them. Gain curves, at least as models of gamete inputs to mating, thus become derived rather than primordial elements of a sex allocation model.

## 6 A Marginal Efficiency Argument

Lloyd (1984) developed a sex‐allocation model for hermaphroditic plants using many standard elements introduced by Charnov (1982). An individual's reproductive resources are divided into a fraction *x* devoted to male function with the remainder 1−*x* devoted to female function. A focal plant, designated by subscript *i*, has a male allocation *x*_*i*_ and fertilizes a fair competitive share of all the ovule fertilizations that take place. If each plant competes in a mating group of *K* pollen donors, then the pollen produced by the focal plant from its allocation *x*_*i*_ competes with the *Kx*_*j*_ pollen grains produced by competitors with male allocation *x*_*j*_. The focal plant's fair competitive share of ovule fertilizations in its mating group is then *x*_*i*_/(*Kx*_*j*_).

The focal plant's female allocation 1—*x*_*i*_ allows the production of (1−*x*_*i*_)*n* seeds, of which a fraction *r* germinate, grow, and survive to maturity, so that the plant's female fitness is (1−*x*_*i*_)*nr*. The *K* other plants in the mating group with male allocation *x*_*j*_ produce *K*(1−*x*_*j*_)*nr* seeds. These seeds represent the ovule fertilizations for which the focal plant competes. The focal plant's fitness through both sex functions can therefore be written as follows:

(3) $$w_{i}=\left(1-x_{i}\right)\mathrm{nr}+\frac{x_{i}}{Kx_{j}}K\left(1-x_{j}\right)\mathrm{nr}$$

(Lloyd 1984, 284). Equation (3) leads to an evolutionarily stable strategy (ESS) allocation at x* = ½.

[There is a subtle but important distinction that can be drawn between two interpretations of *K* in Equation (3). It could represent either the size of the entire population, or the size of the mating subsets into which the population is divided. If the former, the population is well‐mixed for breeding purposes. If the latter, then a focal plant competes with *K* pollen donors for its male success but competes at a population‐wide scale for female fitness through seed production, dispersal, germination, and establishment. The two different scales of competition for male and female fitness lead to a female‐biased sex allocation at an evolutionary equilibrium when *K* is small (Charnov 1980). A useful discussion of these two interpretations of *K* is provided by Fromhage and Kokko (2010, appendix A). Lloyd 1984 uses both interpretations, first in a gain curve model in which *K* is the population size, and later in a plant adaptation of Charnov's 1980 model where *K* represents a mating subgroup. Here I address only the gain curve model with its implicit population‐wide meaning of *K*].

Equation (3) is a Shaw‐Mohler equation in which population fitness has been specified as *W* = *K*(1—*x*_*j*_)*nr*, which may be more apparent upon algebraic rearrangement to a Shaw‐Mohler form with the structure of Equation (2):

(4) $$w_{i}=\left(1-x_{i}\right)\mathrm{nr}+\frac{x_{i}}{Kx_{j}}K\left(1-x_{j}\right)\mathrm{nr}=\left[\frac{\left(1-x_{i}\right)\mathrm{nr}}{K\left(1-x_{j}\right)\mathrm{nr}}+\frac{x_{i}}{Kx_{j}}\right]K\left(1-x_{j}\right)\mathrm{nr}.$$

Equation (4) presents a problem of interpretation. The term *K*(1−*x*_*j*_)*nr* is equivalent to *F* in the denominator of the competitive share ratio *f*_*i*_/*F* of Equation (2), but it is also equivalent to *W* in Equation (2). That is, the same term means both female inputs to the mating arena and fitness outcome in Equation (4). The structure of Equation (4) has been used repeatedly in sex allocation models (e.g., Morgan 1992; Klinkhamer and de Jong 2002; Rademaker and de Jong 2000; Zhang and Jiang 2002; Wiernasz and Cole 2009), with the same incompatibility of meaning. Indeed, it has become a standard way to employ the Shaw‐Mohler equation (Campbell 2000).

Having derived Equation (3), Lloyd (1984) then follows Charnov (1982) in replacing the terms for pollen and seed production with power‐function gain curves. Maternal fitness for the focal plant becomes (1−*x*_*i*_)^*z*^ *nr*, and its competitive share of pollen success becomes *x*_*i*_^*y*^/(*Kx*_*j*_^*y*^). The Shaw‐Mohler form for fitness of the focal plant, on the assumption that *W* = *K*(1−*x*_*j*_)^z^ *nr*, is then

(5) $$w_{i}={\left(1-x_{i}\right)}^{z}\mathrm{nr}+\frac{x_{i}^{y}}{Kx_{j}^{y}}K{\left(1-x_{j}\right)}^{z}\mathrm{nr}=\left[\frac{{\left(1-x_{i}\right)}^{z}}{K{\left(1-x_{j}\right)}^{z}}+\frac{x_{i}^{y}}{Kx_{j}^{y}}\right]K{\left(1-x_{j}\right)}^{z}\mathrm{nr}$$

(Lloyd 1984, 286). Equation (5) leads to an ESS at x* = *y*/(*y* + *z*).

Efficiency is invoked to explain this result: ‘Various biological factors may influence the curvature of either fitness curve…if an increase in pollen makes pollen donation less “efficient” (*y* < 1), the proportional allocation to seeds is increased. If the “efficiency” of a function is dependent on the level of investment in that function, the ESS allocation depends on *the way in which the “efficiency” changes*, and not on the overall “efficiency”’ (Lloyd 1984, 286, italics in the original). *Efficiency* is not defined in this passage or elsewhere, but the description makes sense if we take efficiency to mean marginal efficiency. Under a power‐function gain curve *x* ^*y*^, marginal efficiency is *yx* ^*y*–1^, which decreases as investment *x* increases for any exponents 0 < *y* < 1. The ESS allocation in the model, x* = *y*/(*y* + *z*), ‘depends on *the way in which the “efficiency” changes*,’ that is, on the values of the gain curve exponents. Elsewhere, as well, Lloyd equated saturating gain curves to decreasing efficiency (Lloyd 1983, 528), a description that accords with marginal efficiency.

## 7 Marginal Efficiency Meets the Fisher Condition

Marginal efficiency is said to have the following effect: ‘if an increase in pollen makes pollen donation less “efficient” (*y* < 1), the proportional allocation to seeds is increased’ (Lloyd 1984, 286). This is true according to the calculated ESS allocation, x* = *y*/(*y* + *z*), but Lloyd's conclusion has not taken into account how the Fisher condition restricts the possible values of *y* and *z*. In order to consider the Fisher condition, male and female fitness under x* must be equated. If gain curves represented a portion of the fitness of entirely male and female individuals, as Charnov (1982) definition put is, then at an ESS equilibrium at which all members of the population have the same optimum allocation, every individual's male fitness (x*)^*y*^ must necessarily equal its female fitness (1−x*)^*z*^. Substitution of the ESS solution leads to the requirement

(6) $${\left(\frac{y}{y+z}\right)}^{y}={\left(1-\frac{y}{y+z}\right)}^{z}.$$

The only solution to Equation (6) is *y* = *z*. Thus, the Fisher condition imposes equal gain curve exponents on the model of Lloyd (1984), at which point the optimal sex allocation is fixed at x* = *y*/(*y* + *z*) = ½. The claim that decreasing marginal efficiency of a saturating male gain curve favors an increased allocation to seeds (Lloyd 1984, 286) would therefore be incorrect.

We might, however, note that Lloyd's (1984) model specifies that each of *K* plants contributes (1−*x*)^z^ *nr* seeds that survive to maturity as adult plants. If we take (x*)^*y*^ as individual male fitness and (1−x*)^z^ *nr* as individual female fitness at evolutionary equilibrium, male fitness is measured on a scale of 0 to 1 and female fitness on a scale of 0 to *nr*. Equating these two would be like comparing a temperature measured on the Fahrenheit scale with a temperature measured on the Celsius scale. We would not want to misinterpret the direction of a thermal gradient between 40°F and 20°C just because 40 > 20. The male fitness scale could be converted to the female scale by multiplying *x* ^*y*^ by *nr*, so that the Fisher condition at the ESS becomes (x*)^*y*^ *nr* = (1−*x*)^z^ *nr*. Substitution of *y*/(*y* + *z*) for x* then yields Equation (6) and the constraint that *y* must equal *z*.

Finally, we might note that while the female gain curve is an immediate component of female fitness, *K*(1−*x*)^z^ *nr*, the male gain curve does not indicate fitness per se but rather participates in a ratio, *x*_*i*_^*y*^/(*Kx*_*j*_^*y*^), that apportions total female fitness to the male fitness of individual *i*. At an ESS *x*_*i*_ = *x*_*j*_ = x*, this ratio necessarily equals 1, and so mean individual male fitness equals mean individual female fitness. The Fisher condition seems satisfied. But this solution creates problems for interpretation of the biological meaning of the male gain curve, because any function, even biologically meaningless functions, will satisfy the Fisher condition. For example, a male gain curve (−*x*)^*y*^ leads to the same ESS, x* = *y*/(*y* + *z*), and appears to satisfy the Fisher condition constructed as

(7) $$\frac{{\left(-x^{*}\right)}^{y}}{K{\left(-x^{*}\right)}^{y}}K{\left(1-x^{*}\right)}^{z}\mathrm{nr}={\left(1-x^{*}\right)}^{z}\mathrm{nr},$$

But what can (−*x*)^y^ mean as a gain curve, since it produces imaginary numbers for most values of *x* and *y*? A Fisher test that can be satisfied by any gain curve, even at the expense of biological meaning, is an indictment not of the Fisher condition but of gain curves and the way they are used as substitutes for the terms of the Shaw‐Mohler equation (Burd 2024, 2026).

The role Lloyd (1984) gives to marginal efficiency, controlled, as it is, by the exponent y, therefore seems unlikely, given the difficulties raised with the Fisher condition. However, ‘gain’ curves will not contravene the Fisher condition if they are used in a Shaw‐Mohler equation to mean the numerical inputs to a mating process rather than fitness outcomes. Harder and Johnson (2025) proposed such a model, which is examined below. It may seem surprising that a simple change in the meaning of gain curves frees the model from entanglement with the Fisher condition, but, as noted above, the mathematical operations of a sex allocation model are not the issue, but the biological lessons we draw, and those lessons depend on the meaning of the terms. Even with satisfaction of the Fisher condition, the Harder and Johnson (2025) model must confront the distinction between fractional and marginal efficiency.

## 8 Marginal Efficiency of Inputs to Mating

Pollen‐ovule ratios in flowering plants tend to be negatively related to fractional pollination efficiency in interspecific comparisons (Gong and Huang 2014; Harder and Johnson 2023). This is the empirical pattern that Harder and Johnson (2025) wished to explain with an argument about the effect of marginal pollination efficiency on sex allocation (and thus on pollen‐ovule ratios, on the plausible presumption that pollen‐ovule ratios will follow the same pattern as sex allocation generally). It emerges that fractional efficiency and marginal efficiency have opposite effects on sex allocation in the model they produced.

Harder and Johnson (2025) replaced Lloyd's power‐function gain curves with a hyperbolic function representing pollen export, *E*(*x*) = *E*_max_ *x*/(*x* + *d*), and a linear function representing seed production, (1−*x*)*s*. *E*_max_ is simply a scaling factor for the hyperbolic pollen export function; *d* is the controlling parameter affecting the shape of the export curve (Figure 2); and *s* is the maximum number of seeds that a plant can produce if it devotes all reproductive resources to female function (i.e., if male allocation *x* = 0). Marginal efficiency, labeled *e*', is explicitly defined as the derivative of the pollen export curve, *e*' = *d*/(*x* + *d*)^2^ (Harder and Johnson 2025, Equation 6). [The derivative would actually be *E*_max_ *d*/(*x* + *d*)^2^. In their figure 1, Harder and Johnson 2025 specify *E*_max_ = 0.2. The graphical presentation of marginal efficiency is scaled to this value in panel 1d of their figure, but not in panel 1b. This ambiguity in the definition affects the magnitude of efficiency, but as *E*_max_ is a simple scaling factor, its presence or absence does not affect the qualitative patterns that emerge from the model].

Given the pollen export and seed production functions, the fitness of a focal plant with allocation *x*_*i*_ in a large population with common allocation *x*_*j*_ is

(8) $$w_{i}=\left(1-x_{i}\right)s+\frac{E_{\max}x_{i}/\left(x_{i}+d\right)}{E_{\max}x_{j}/\left(x_{j}+d\right)}\left(1-x_{j}\right)s=\left(1-x_{i}\right)s+\frac{x_{i}\left(x_{j}+d\right)}{x_{j}\left(x_{i}+d\right)}\left(1-x_{j}\right)s$$

Harder and Johnson (2025, Equation 7). Equation (8) leads to an ESS allocation at x* = (*d* + *d* ^2^)^½^ – *d*.

Because *E* represents the input of pollen grains to the pollination arena, this ESS solution does not run afoul of the Fisher condition in the way Lloyd's (1984) solution does. The numbers of exported pollen grains and seeds need not be commensurate. Indeed, pollen grains typically exceed ovule number (and thus seed number) by two or three orders of magnitude (Erbar and Langlotz 2005).

The ESS allocation x*, indicated by an open circle on each curve in Figure 2, increases as parameter *d* increases. The marginal efficiency at the ESS, *e*'(x*) = *E*_max_/(1 + *d*), is a decreasing function of *d*. The pattern of marginal efficiency can be visualized in Figure 2 by the slopes of the tangents to the different export curves at each ESS; x* and *e*'(x*) are negatively related (Table 1). Therefore, ‘plants in populations with lower mean allocation to the production of pollen and its dispersal should realize greater mean pollen‐export efficiency than those in populations that allocate relatively more to male function’ (Harder and Johnson 2025, 42). The catch is that pollen export efficiency in this context means marginal efficiency. The empirical pattern that the model is meant to explain (Harder and Johnson 2023) is based on fractional efficiency. What are the predictions regarding fractional efficiency?

**TABLE 1:** Marginal and fractional efficiency of pollen export at the ESS allocation x* in the model of Harder and Johnson (2025). The elements of this table correspond to Figure 2.

*d*	x*	*E*(x*)^a^	*e*'(x*)^b^	Pollen production^a^	Fractional efficiency
0.1	0.232	0.698	0.909	2.317	0.301
0.5	0.366	0.423	0.667	0.732	0.578
1	0.414	0.293	0.5	0.414	0.707
2	0.449	0.184	0.333	0.225	0.817

^a^ Values indicate a multiple of *E*_max._

^b^ Values indicate a multiple of *E*_max_ per unit male allocation.

In order to assess fractional efficiency, pollen production must be known. The export curve *E* was proposed without any reference to pollen production, but a minimum allowable production can be inferred, as in Figure 1. The equivalent of P0 in Figure 1—that is, linear production that provides sufficient pollen to account for an entire gain curve—is given in Figure 2 by the tangent to the export curve *E* at *x* = 0. The hyperbolic export curves in Figure 2 have the fortunate mathematical property that the slope of the tangent is finite at *x* = 0. They have the less fortunate property that the slope varies as *d* varies, that is, the rate at which resource investment is converted to pollen production depends on how the pollen then gets dispersed. The biological meaning of this property would need some interpretation. Moreover, changes in parameter *d* affect both pollen production and efficiency rather than efficiency alone, somewhat like an experiment with two uncontrolled variables. But if we accept this property, then fractional efficiency at the ESS for each curve is the ratio of pollen export, *E*(x*), to pollen production at x*. Fractional efficiency can be visualized in Figure 2 as the vertical height of an export curve at the ESS, shown as the open circles, divided by the vertical height on the corresponding production line directly above the ESS. Fractional efficiency is an increasing function of *d*, while marginal efficiency is a decreasing function of *d* (Table 1).

## 9 The Effect of a Stable Pollen Production Pattern

The shortcomings and ambiguities of the gain curve approach to reproductive efficiency are easily repaired by establishing explicit functions for the production of mating agents and for their reproductive efficiency. Gamete export curves emerge as derived products of these primordial functions, rather than being a priori stipulations.

Suppose a plant could produce *P*_max_ pollen grains if it devoted all its reproductive resources to male function, and *xP*_max_ pollen grains if it devotes a fraction *x*. Similarly, it could produce *O*_max_ ovules if all resources go to female function, and (1−*x*)*O*_max_ ovules if male allocation is *x*. It will not matter for the argument whether ovule production or eventual seed production is modeled, provided we assume that the maturation of ovules to seeds occurs randomly with respect to the sex allocation of the pollen parents (the sex allocation of the maternal parent is *x*_*j*_ for all ovules of plant *j*).

**FIGURE 3:**
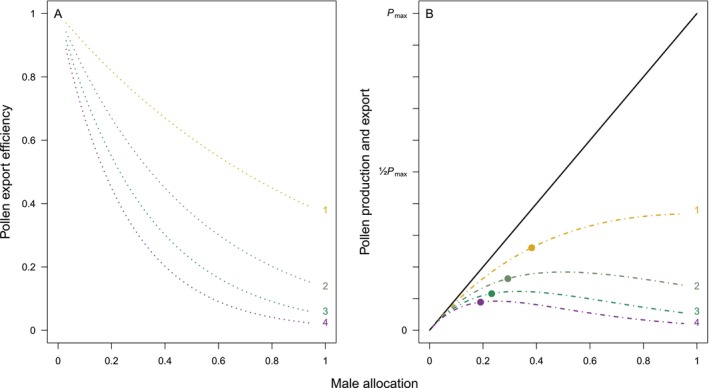
Example of a priori definition of pollination efficiency and pollen production functions, and the pollen export curves they generate. (A) Pollen export efficiency, *g*(*x*), in relation to male allocation, depicted here as a negative exponential function, *g*(*x*) = exp(−*bx*) for a positive parameter *b* (indicated to the right of the curves). (B) Pollen production and export, scaled to the maximum number of pollen grains an individual can produce, *P*_max_. Production, *xP*_max_, is indicated by the continuous line. Pollen export, *xP*_max_ *g*(*x*), is shown as four interrupted curves corresponding to the four efficiency curves in panel A (values of parameter *b* shown to the right of the curves). ESS allocations derived from Equation (11) are shown as filled circles for each export curve.

Now suppose that the fraction of pollen exported by a plant to conspecific flowers is a function of the amount of pollen produced. Denote this fraction *g*(*x*). It corresponds to the dimensionless, fractional sense of efficiency. Clearly, *g*(*x*) cannot exceed 1 over the domain of possible allocations, as values greater than 1 would imply that more pollen is exported than was produced. Nor can *g*(*x*) be negative. But it will not be necessary to specify *g*(*x*) any further, other than to note that, typically, *g*(*x*) would be a decreasing function of *x*. Several empirically established mechanisms tend to reduce pollen export efficiency as male investment rises, including diminishing attractiveness of inflorescence size to pollinators (Lau et al. 2008; Karron and Mitchell 2012) and increased grooming by pollinators when greater pollen loads are deposited (Harder and Johnson 2025). The example in Figure 3 uses a specific function for *g*(*x*), but there is no need to give a specific form to *g*(*x*) in order to derive a meaningful solution.

The number of pollen grains exported from a plant will be *xP*_max_ *g*(*x*). This is pollen export equivalent to the function *E* of Harder and Johnson (2025), but rather than having to infer pollen production from the curve, as in Figure 2, the production function is specified and fixed, *xP*_max_ (Figure 3B).

If plant *i* is a focal mutant in a large population of plants using sex allocation *x*_*j*_, its fitness in Shaw‐Mohler form is

(9) $$w_{i}=\frac{\left(1-x_{i}\right)O_{\max}}{\left(1-x_{j}\right)O_{\max}}+\frac{x_{i}P_{\max}g\left(x_{i}\right)}{x_{j}P_{\max}g\left(x_{j}\right)}.$$

Equation (9) shares the limitations of the Shaw‐Mohler equation generally. It does not specify aggregate population fitness and would not represent any process that translates pollen and ovule abundance to aggregate fitness if it were specified. But the Shaw‐Mohler equation captures the action of frequency‐dependent selection, and that suffices for the present purpose.

Taking the derivative of Equation (9), and setting *dw*_*i*_/*dx*_*i*_ = 0 and *x*_*i*_ = *x*_*j*_ = *x* to find ESS candidates yields

(10) $${\left.\frac{dw_{i}}{dx_{i}}\right|}_{x_{i}=x_{j}=x}=\frac{1}{x}-\frac{1}{1-x}+\frac{g^{\prime }\left(x\right)}{g\left(x\right)}=0,$$

in which *g*'(*x*) represents the derivative of *g*(*x*). Equation (10) is satisfied if

(11) $$\frac{g^{\prime }\left(x\right)}{g\left(x\right)}=\frac{1}{1-x}-\frac{1}{x}.$$

**FIGURE 4:**
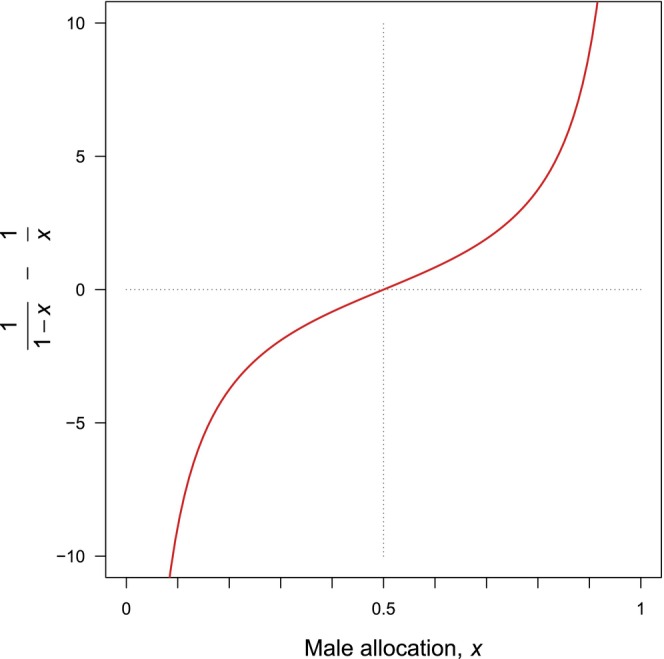
Behavior of 1/(1−*x*) – 1/*x*, the quantity on the right‐hand side of Equation (11).

The biological meaning of Equation (11) is readily interpreted. If pollen export efficiency is a decreasing function of male allocation, then the derivative *g*'(*x*) will be negative. The equality in Equation (11) will be satisfied only if the quantity on the right‐hand side of Equation (11) is also negative. Figure 4 shows that the right‐hand side is an increasing function of *x* and has negative values when *x* is less than ½ (female‐biased allocation). If pollen export efficiency is a constant fraction of pollen production, the derivative *g*'(*x*) = 0 and only *x* = ½ then satisfies Equation (11). If, contrary to expectation, pollen export efficiency increases as male allocation increases, then *g*'(*x*) > 0, and male allocation *x* must exceed ½ (male‐biased allocation) to satisfy Equation (11).

The example in Figure 3 can be used to illustrate the ESS requirement in Equation (11). Pollen export efficiency is taken to be a declining exponential function *g*(*x*) = exp(−*bx*) (Figure 3A), and so *g*'(*x*)/*g*(*x*) = −*b*. Making this substitution into Equation (11) and solving for *x* yields the ESS value x* = [*b* + 2 – (*b* ^2^ + 4)^½^]/(2*b*). Greater fractional efficiency at the ESS (i.e., ESS points lying closer to the production line) also involves higher ESS male allocation (Figure 3B). This positive relationship between x* and fractional efficiency (Table 2) is qualitatively similar to the corresponding relationship in the model of Harder and Johnson (2025). However, there is also a positive relationship between x* and the marginal efficiency of these pollen export curves at x* (Table 2), opposite to the negative relationship found by Harder and Johnson (2025) for their model (Table 1). The difference occurs because pollen production is not a fixed function in their model, as it is in Figure 3B, but must instead be inferred from the export curve (Figure 2).

**TABLE 2:** Marginal and fractional efficiency of pollen export at the ESS allocation x* in the model with a priori definition of the pollen production function. The elements of this table correspond to Figure 3.

b	x*	Pollen export^a^	Marginal efficiency^b^	Pollen production^a^	Fractional efficiency
1	0.382	0.261	0.422	0.382	0.683
2	0.293	0.163	0.231	0.293	0.557
3	0.232	0.116	0.151	0.232	0.498
4	0.191	0.089	0.110	0.191	0.466

^a^ Values indicate a multiple of *E*_max._

^b^ Values indicate a multiple of *E*_max_ per unit male allocation.

## 10 Discussion

As we pry open the concept of reproductive efficiency, we discover several fundamental features of gain curves and the Shaw‐Mohler equation:

1. Gain curves by themselves tell us nothing about fractional reproductive efficiency because they tell us nothing about the pattern of production of the entities that acquire fitness. This is true even if a gain curve is interpreted as the entry of gametes into a mating arena rather than the fitness outcome.
2. Biologically allowable linear production patterns inferred from power‐function gain curves imply biologically impossible rates of conversion of resources into mating agents (Figure 1 and associated text). Nonlinear production patterns can escape this problem, but the changing cost per mating agent produced implies economies or diseconomies of scale that would need biological justification.
3. The Shaw‐Mohler equation represents an individual's fair competitive share of aggregate population fitness based on the abundance of male and female mating agents it produces. Competitive share is expressed in a ratio for male function and another ratio for female function, as in Equation (1). The scaling need not be identical because the ratios of Equation (1) represent competitive share *within* each sex function. But we must recognize that the terms in these ratios represent the abundance of male and female entities and not the outcome of their activity. Substitution of fitness functions (gain curves) for the abundance terms in Equation (1) leads easily to violation of the Fisher condition, while adherence to the Fisher condition severely restricts gain curves, often to linear gains (Burd 2026). Charnov (1982) thus erred in substituting fitness gain curves for the abundance terms of the Shaw‐Mohler equation (Burd 2024), and most sex allocation models based on the Shaw‐Mohler equation have repeated the error.
4. The Shaw‐Mohler equation contains no representation of how the abundance of mating agents leads to total population fitness. Indeed, total population fitness need not even be specified, as in Equation (1), and if it is, as in Equation (2), the specification has no effect on the calculated ESS. This need not pose a problem if our only interest is in the action of frequency‐dependent selection. But the process of translation of mating agents into eventual fitness is at the core of reproductive efficiency. The Shaw‐Mohler equation is thus an inherently poor tool for examining the effect of efficiency on sex allocation, and for other phenomena such as sperm limitation of egg fertilization (Levitan 1998; Yund 2000) or pollen limitation of seed set (Burd 1994b; Rodger et al. 2021).

A conceptually simple (albeit mathematically more elaborate) alternative to gain curves and the Shaw‐Mohler equation is to represent mating interactions as a dynamic process in which production, entry to a mating arena, and mating of male and female agents are explicitly represented by separate rates. Pirrie and Ashby (2021) were the first to use this approach, which verified the long‐established conclusion of sex allocation theory that sex differences in mortality had no effect on sex allocation provided the mortality occurs following the period of parental resource investment in offspring (Shaw and Mohler 1953; Leigh 1970; Charnov 1982; West 2009; Gardner 2023). Bochynek and Burd (2024) used the same mathematical approach to examine the effect of post‐anthesis loss of pollen (i.e., fractional pollination efficiency) and also found that different loss rates for pollen and ovules had no effect on sex allocation (although very small mating group sizes depressed the optimal allocation to male function, just as small mating groups affected male investment due to local mating competition (LMC) in the barnacle model of Charnov 1980, 1982). The structure of these models is superior to the Shaw‐Mohler equation in that they represent the processes that connect production of mating agents to the fitness they eventually achieve. This structure can accommodate the nonlinear effects that gain curves are meant to represent.

Reproductive efficiency (in the common, fractional sense) may be high or low, but provided it is constant, so that *g*'(*x*) = 0 in Equation (9), the ESS allocation will be x* = ½ (Figure 4). This result accords with longstanding conventional understanding of sex allocation theory. If fractional efficiency declines as a function of allocation, however, then efficiency and allocation at an ESS should be positively related (Table 2). Applied to pollen‐ovule ratios, this result predicts that species with low pollination efficiency should have low male allocation (and therefore low pollen‐ovule ratios, to the extent pollen and ovule numbers mirror sex allocation in general); high pollination efficiency should be accompanied by greater pollen investment. The empirical interspecific pattern among angiosperms is the opposite, a negative relationship between dimensionless pollination efficiency and pollen‐ovule ratio (Erbar and Langlotz 2005; Gong and Huang 2014; Harder and Johnson 2023). Efficiency is therefore unlikely to be the direct cause of this relationship. Some third factor must affect both efficiency and pollen‐ovule ratios but in opposite ways, or be an intermediary between efficiency and pollen‐ovule ratios in a way that negates the theoretically expected effect of allocation‐dependent efficiency on pollen‐ovule ratios.

A possibility for such a third factor is efficiency‐dependent local mating competition (LMC) (Bochynek and Burd 2024; Burd 2025). Efficient pollen transfer to stigmas is often a product of precise placement of pollen on animal vectors as a result of petal fusion and zygomorphy (Grant 1994; Citerne et al. 2010; Yoder et al. 2020; Stewart et al. 2022), deep corolla tubes (Nilsson 1988; Montgomery and Rathcke 2012), or other features of floral morphology that restrict pollen deposition to specific areas on a pollinator body (Flanagan et al. 2009; Muchhala and Thomson 2012). Precise pollen placement from source flowers would tend to enrich stigmatic loads of recipient flowers with kin pollen. Any other mechanism that increases the efficiency of pollen export, even as simple a factor as an abundant pollinator fauna and high floral visitation rates, would tend to flood a neighborhood of mating opportunities with repeated pollen deposition from any given donor. Pollen grains from the same donor then compete with each other as fathers of embryos that compete for maternal resources for seed maturation. The LMC that would be created by either mechanism would tend to favor reduced male allocation (Hamilton 1967; Charnov 1982; West 2009), thus countering the theoretically expected effect of fractional efficiency on its own (Burd 2025).

Stigmatic deposition of pollen loads enriched in kin pollen would tend to leave an empirical trace: correlated paternity of seeds within fruits. Repeated efficient pollen donation to a restricted neighborhood of recipients would tend to elevate the level of correlated paternity among seeds of different fruits on the same maternal plant. Both types of correlated paternity seem to be common (Ritland 1989; Hardy et al. 2004; Silva et al. 2011; Dorken and Perry 2017; Diaz‐Martin et al. 2023). In contrast, wind pollination tends to disperse pollen more widely than animal pollinators do, resulting in lower genetic differentiation among populations than in animal‐pollinated species (Wessinger 2021) and low rates of correlated paternity (Colabella et al. 2014; Ortego et al. 2014; Kikuchi et al. 2015). Better dispersal reduces LMC and favors greater male allocation (Fromhage and Kokko 2010). Accordingly, wind pollination is associated with high pollen‐ovule ratios (Cruden 2000; Erbar and Langlotz 2005; Michalski and Durka 2009). Thus, some existing evidence is consistent with efficiency‐dependent LMC, and the hypothesis is amenable to further testing of the prediction that species with relatively high rates of correlated paternity in their mating structure will have relatively low pollen‐ovule ratios.

What about marginal efficiency? Harder and Johnson (2025) predict that greater marginal efficiency of pollination will be associated with lower pollen‐ovule ratios. They apply their result to the empirical relationship between fractional efficiency and pollen‐ovule ratios, but this is a misapplication. Testing the Harder and Johnson (2025) prediction awaits an appropriate interspecific data set combining empirical measurements of marginal efficiency (e.g., Figure 1) with pollen‐ovule ratios.

## 11 Conclusion

The many contrivances of animals and plants that increase reproductive efficiency show that higher fractional efficiency is favored by natural selection. For example, broadcast‐spawning marine invertebrates may accumulate dilute but long‐lived sperm over a long period in advance of egg production to enhance mating success of eggs (Pemberton et al. 2003). The intertidal brown alga *Fucus vesiculosus* releases sperm only in calm waters to minimize loss through turbulent water movement (Serräo et al. 1996). Flowering plants have many adaptations of floral structure to promote accurate pollen deposition on pollinator bodies, as noted above, and may simply evolve new pollinator relationships to overcome inefficient pollination (Dellinger et al. 2021; Duncan et al. 2024). Greater efficiency improves individual competitive ability and may improve mean fitness if reproductive success in a population is mate‐limited. Selection in favor of reproductive efficiency is not in doubt.

At issue is whether reproductive efficiency in turn affects sex allocation. The models examined here suggest it can if the degree of efficiency itself varies with sex allocation. But the direction of the effect depends on the type of efficiency considered and how production of mating agents is represented.

In examining the relationship of reproductive efficiency to sex allocation, the limitations of two fundamental tools of sex allocation theory, gain curves and the Show‐Mohler equation, come into focus. Gain curves have been asserted a priori without reference to the production of mating agents. Retroactively inferring production from gain curves reveals new problems because the implied number of successful matings cannot exceed the number of mating agents produced. The Shaw‐Mohler equation is designed to embody frequency‐dependent selection if the terms of the equation represent the numbers of male and female entities present in a mating arena. Substitution of male and female fitness gain curves for these numbers leaves the Shaw‐Mohler equation susceptible to violations of the Fisher condition. These shortcomings are known (Burd 2024, 2026) but are seen especially clearly through the lens of reproductive efficiency.

## Author Contributions

**Martin Burd:** conceptualization (equal), writing – original draft (equal), writing – review and editing (equal).

## Conflicts of Interest

The author declares no conflicts of interest.

## Data Availability

No external data were used in this manuscript other than digitized data from a published source used to create Figure 1.
